# Delta-Like Canonical Notch Ligand 1 in Patients Following Liver Transplantation—A Secondary Analysis of a Prospective Cohort Study

**DOI:** 10.3390/diagnostics10110894

**Published:** 2020-10-31

**Authors:** Sebastian O. Decker, Dagmar Hildebrand, Thomas Bruckner, Christoph Lichtenstern, Klaus Heeg, Markus A. Weigand, Thorsten Brenner, Florian Uhle

**Affiliations:** 1 Department of Anesthesiology, Heidelberg University Hospital, Im Neuenheimer Feld 420, 69120 Heidelberg, Germany; christoph.lichtenstern@med.uni-heidelberg.de (C.L.); markus.weigand@med.uni-heidelberg.de (M.A.W.); Thorsten.Brenner@uk-essen.de (T.B.); florian.uhle@med.uni-heidelberg.de (F.U.); 2 Medical Microbiology and Hygiene, Centre for Infectious Diseases, Heidelberg University Hospital, Im Neuenheimer Feld 324, 69120 Heidelberg, Germany; dagmar.hildebrand@med.uni-heidelberg.de (D.H.); klaus.heeg@med.uni-heidelberg.de (K.H.); 3 Institute of Medical Biometry and Informatics, University of Heidelberg, Im Neuenheimer Feld 130, 69120 Heidelberg, Germany; bruckner@imbi.uni-heidelberg.de; 4 Department of Anesthesiology and Intensive Care Medicine, Essen University Hospital, Hufelandstraße 55, 45147 Essen, Germany

**Keywords:** DLL1, liver transplantation, bacterial infections, complicated course

## Abstract

Opportunistic bacterial infections are dreaded risks in patients following liver transplantation (LTX), even though patients receive an antibiotic prophylaxis. The timely recognition of such an infection may be delayed, as culture-based diagnostic methods are linked with a relevant gap in performance. We measured plasma concentrations of Delta-like canonical Notch ligand 1 (DLL1) in 93 adult patients at seven consecutive time points after liver transplantation and correlated the results to the occurrence of culture-proven bacterial infection or a complicated clinical course (composite endpoint of two or more complications: graft rejection or failure, acute kidney failure, acute lung injury, or 90-day mortality). Patients exhibited elevated plasma concentrations after liver transplantation over the whole 28 d observation time. Patients with bacterial infection showed increased DLL1 levels compared to patients without infection. Persistent elevated levels of DLL1 on day 7 and afterward following LTX were able to indicate patients at risk for a complicated course. Plasma levels of DLL1 following LTX may be useful to support an earlier detection of bacterial infections in combination with C-reactive protein (CRP) and procalcitonin (PCT), or they may lead to risk stratification of patients as a single marker for post-operative complications. (Clinical Trial Notation. German Clinical Trials Register: DRKS00005480).

## 1. Introduction

Since the first liver transplantation (LTX) in 1963 [1], the method has been become a routine therapeutic option in patients with end-stage liver diseases (ESLD). Notably, the quality of life of LTX patients has increased through the use of improved immunosuppressive treatment regimens as well as optimized standard care procedures [2,3]. Nevertheless, these improvements are also linked with an elevated risk for bacterial infections in the first year after LTX [4], which may lead to an increased mortality [5]. Therefore, fast and reliable diagnostic tools are needed to detect these infections as early as possible.

Similar to infection detection for septic patients, the most commonly used laboratory parameters for the detection of an infection following LTX are C-reactive protein (CRP) and procalcitonin (PCT) [6,7]. Often used as the gold standard in septic patients, PCT shows different kinetics in patients following LTX, with highly elevated levels directly after transplantation without having relevant infections [8,9]. Besides CRP, which has a low specificity after LTX due to an existing sterile inflammation, interleukin (IL)-6 might be a valuable marker for infectious complications [8], but it is often claimed to be unspecific. Therefore, the usefulness of these biomarkers in clinical routine, especially for complex transplant patients at inherently high risk for infection, is strongly limited.

Delta-like canonical Notch ligand 1 (DLL1), a transmembrane protein of the Delta/Jagged family and ligand of Notch receptors, was found to be upregulated in primary human monocytes in response to an in vitro bacterial infection [10], subsequently resulting in high concentrations of its cleavage product, soluble DLL1 in the cell supernatant. Importantly, in the plasma of septic patients caused by an underlying bacterial infection, DLL1 was also found to be elevated compared to different control groups (trauma patients, patients following extensive visceral surgery, and healthy volunteers), rendering it a robust biomarker for the diagnosis of severe bacterial infection [11]. However, its diagnostic value in patients following LTX is unknown.

Therefore, the aims of this secondary analysis are to evaluate the performance of DLL1 to diagnose bacterial infection as well as to predict a complicated clinical course of patients following liver transplantation compared to standard biomarkers such as PCT or CRP.

## 2. Materials and Methods

### 2.1. Study Design

Samples from an already published study [12] were examined in terms of a secondary analysis. The primary study as well as the research presented here were approved by the local ethics committee (Ethics Committee of the Medical Faculty of Heidelberg, Trial Code No. S-098/2013/German Clinical Trials Register: DRKS00005480) and were performed in accordance with the ethical standards of the institutional research committee and with the 1964 Helsinki declaration and its later amendments or comparable ethical standards. Blood sampling and corresponding clinical data collection was carried out in the surgical intensive care unit at the Heidelberg University Hospital from 2014 to 2016. Informed consent was given in written form by all participating patients. 93 patients with ESLD after LTX were included in the original study and treaded standardized, according to a published manual [13]. For this secondary analysis, plasma samples of all 93 patients were available. The samples were collected directly after liver transplantation up to day 28 at seven defined timepoints (day (d) 0, d1, d2, d7, d14, d21, d28). All clinical routine data other than plasma samples, including several diseases severity scores and infection markers as well as microbiological results, were available and used for re-analysis. The study was reported according to the recommendations of the STROBE statement (Supplementary Statement S1).

### 2.2. Subgroup Definitions

Patients were grouped into the “Bacterial Infection group” according to the criteria of the American Society of Transplantation published in 2006 [14] and supplemented with plasmatic infections markers as well as clinical signs. An infection was considered in the case of (1) blood culture positivity in combination with elevated infection markers (CRP > 10 mg/dL or PCT > 1 µg/L), acute clinical deterioration (e.g., tachypnea, fever, hypotension (systolic blood pressure < 90 mmHg), shivering) and a need for an antibiotic, (2) a microbiological finding from intraoperative swabs or in fluids out of new established interventional drainages from primary sterile sites in cases of radiologically or surgically diagnosed abscesses in combination with elevated infection markers (CRP > 10 mg/dL or PCT > 1 µg/L) and a need for an antibiotic, or (3) high bacterial burden in pre-existing catheters in combination with acute clinical deterioration (e.g., tachypnea, fever, hypotension (systolic blood pressure < 90 mmHg), shivering), elevated infection markers (CRP > 10 mg/dL or PCT > 1 µg/L) and a need for an antibiotic. In patients with an infection, a new virtual timepoint (V) was created according to the first timepoint of bacterial detection and used for further analyses of DLL1 and routine infection markers. A valid comparison group was built by age- and sex-matching the virtual timepoints to the uninfected patients.

**Figure 1:**
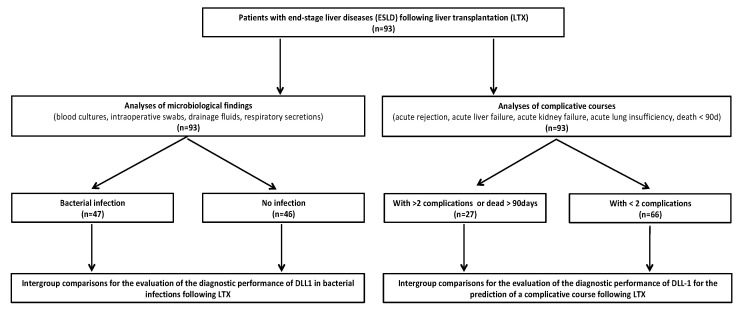
Flow chart of included patients. Abbreviations: ESLD, end-stage liver disease; LTX, liver transplantation.

In a second approach, all patients were screened for the following events within the first 28 days: acute rejection, acute graft failure, acute renal failure, and acute lung insufficiency. An acute rejection was supposed when liver enzymes and parameters for cholestasis increased in combination with a liver biopsy, classified by a score higher than 4 according to the latest version of the Banff criteria [15], leading to the initiation of a corticosteroid pulse therapy. A transplant liver failure was supposed when liver synthesis function decreased acutely in combination with elevated liver enzymes and parameters for cholestasis without signs of an acute rejection. An acute renal failure was defined as a minimum doubling of retentions markers in combination with a worsening urinary production or need for dialysis adapted to the Kidney Disease: Improving Global Outcomes (KDIGO) criteria published in 2012 [16]. Acute lung failure was diagnosed according to the Berlin definition of ARDS (acute respiratory distress syndrome) published by the ARDS Definition Task Force in 2012 [17]. In the case of two or more complications or death within 90 days, a composite endpoint was built and the patients were classified as a “complicated course” group. The corresponding flow diagram of patients’ inclusion is shown in Figure 1.

### 2.3. Measurement of DLL1

Plasmatic DLL1 levels were evaluated using a commercially available ELISA kit (RayBiotech Life, Inc., Norcross, GA, USA) according to the manufacturer’s instructions. All samples were diluted 1:30 (or higher if demanded by the plasma concentration to maintain the measurement within dynamic range) with the supplied Assay Diluent A to minimize interfering matrix effects. Absorbance measurements were performed on an ELx808 microplate reader (BioTek Instruments, Inc., Winooski, VT, USA) with a subsequent automatized calculation of concentrations within the corresponding Gen5 software (BioTek Instruments, Inc., Winooski, VT, USA).

### 2.4. Statistical Analyses

All collected data were recorded in an electronic database (Excel 2019, Microsoft Corp, Redmond, WA, USA) and analyzed using SPSS software (Version 25.0, IBM, New York, NY, USA). Boxplots were drawn using GraphPad Prism 8.3 (GraphPad Software, La Jolla, CA, USA) and assembled with a presentation software (PowerPoint 2019, Microsoft Corp, Redmond, WA, USA). Receiver–operator curves to assess the diagnostic performance were created using SPSS software. After checking the non-normal distribution using the Kolmogorov–Smirnov test, the chi-squared test was used for categorical data and the Mann–Whitney *U*-test was used for continuous data. Data are given as absolute or relative frequencies in the case of categorical data or as median values with first and third quartiles in the case of quantitative data. A multivariate binary logistic regression analysis was established to determine the influence of potential confounders. Therefore, a cutoff value for DLL-1 at day 2 was calculated and a corresponding binary variable was invented. Moreover, another logistic regression was used to evaluate the value of different combinations of infection parameters. Statistical significance was considered when *p* < 0.05. The following symbols of significance were used: *p* < 0.05: *, *p* < 0.01: **, *p* < 0.001: ***.

## 3. Results

### 3.1. Patients’ Characteristics

In total, 93 patients were included in the analysis for this study (Figure 1). Patients’ characteristics as well as details of the perioperative course are presented in Table 1 for patients with or without bacterial infection, while details of patients with or without a complicated course following LTX are pointed out in Table 2.

In total, 47 (50.5%) of the 93 examined patients developed a bacterial infection after LTX. No obvious differences between the characteristics of patients with bacterial infection or without it could be observed. Patients with a bacterial infection showed higher pre-transplant model of end-stage liver disease (MELD) scores and remained longer both in the intensive care unit (ICU) and in the hospital; they were also associated with a significantly higher need for renal replacement therapy (RRT) in the time course as well as a prolonged need for mechanical ventilation. However, there were no significant differences concerning the 28-day and 90-day mortality. Regarding first bacterial detection, infected patients could be clustered in three groups: 21 (44.7%) of the 47 patients had a positive bacterial culture within the first 7 days after LTX, 15 (31.9%) patients showed the first detection between day 8 and 14, and 11 (23.4%) patients had their first finding at day 15 or later on. The following bacterial findings were found (double naming feasible): 35 patients (37.6%) showed a positive bacterial finding within a blood culture, 32 patients (34.4%) revealed bacterial drainage infections, 13 patients (13.9%) presented with pneumonia, and 11 patients (11.8%) had a urinary tract infection. Concerning multidrug-resistant (MDR) bacterial findings, 2 patients (2.1%) were colonized with MRSA, 12 patients (12.9%) showed a colonization with vancomycin-resistant *Enterococcus faecium* (VRE), 3 patients (3.2%) were colonized with multidrug-resistant Gram-negative bacteria (MRGN), and 9 patients (9.7%) showed a simultaneous colonization with MRGN and VRE. Within the 28-day observation period, 8 patients (8.6%) acquired a new VRE colonization, 5 patients (5.4%) acquired a new MRGN colonization, 8 patients (8.6%) developed a positive blood culture with VRE, and 7 patients (7.5%) a positive blood culture with MRGN. Within a multivariate regression analysis, no significant confounders for bacterial infections within our patients were determined (Table 3).

27 of 93 patients (29.0%) experienced two or more relevant complications within the 28 days or died within 90 days after LTX (Table 2). Compared to patients without complications, these patients possessed a significant higher body mass index (BMI) with 25.53 (Q1:23.08–Q3:30.72) and more frequent primary sclerosing cholangitis and non-alcoholic steatohepatitis (NASH) as underlying diseases. The clinical course was heavily impacted in this subgroup, with a prolonged need for mechanical ventilation, a significant higher incidence of rejections, a prolonged ICU and hospital stay, and a reduced 90-day survival (Table 2). Within a multivariate analysis, only the posttransplant need for dialysis was observed as a risk factor for the occurrence of two or more complications (Table 4). Unsurprisingly, DLL-1 levels showed significant elevated levels in patients with a need for posttransplant dialysis (Supplementary Table S1).

### 3.2. DLL1 and PCT for the Detection of Bacterial Infection after LTX

**Figure 2:**
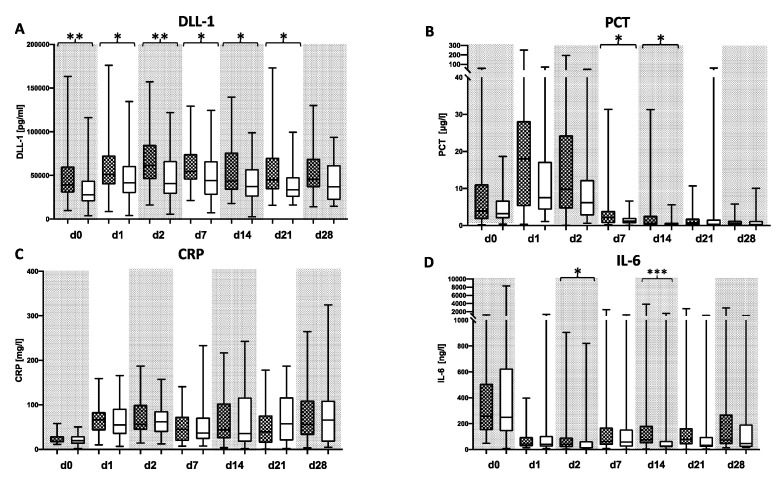
Delta-like Canonical notch ligand 1 (DLL1) for the recognition of a bacterial infection following liver transplantation (LTX). Plasma concentrations of (**A**) Delta-like Canonical notch ligand 1 (DLL1), (**B**) procalcitonin (PCT), (**C**) C-reactive protein (CRP), and (**D**) interleukin (IL-)6 were measured in patients following LTX with a bacterial infection (black-checkered box) or without any bacterial findings (white box). Plasma samples were collected directly after LTX and within the following 28 days afterward on the indicated day. Data presentation: box plots with median, 25th percentile and 75th percentile in the box, as well as with the 10th and 90th percentiles at the end of the whiskers. Symbols of significance: *p* < 0.05 *, *p* < 0.01 **, *p* < 0.001 ***.

**Figure 3:**
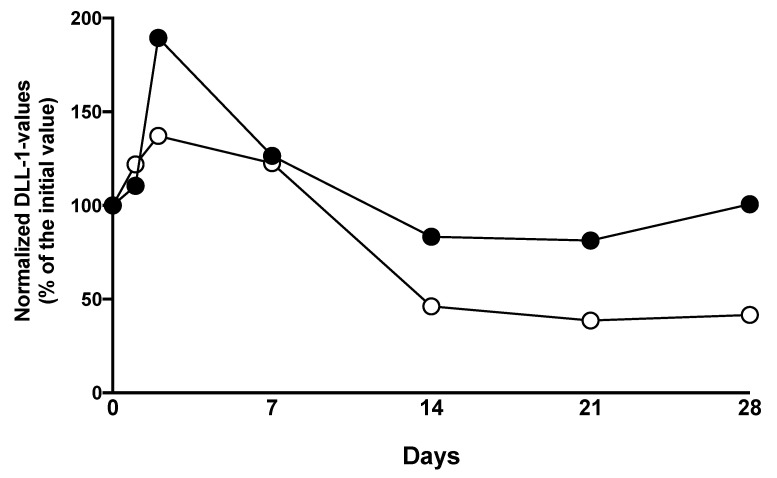
Percentage changes of Delta-like Canonical notch ligand 1 (DLL1) for the recognition of a bacterial infection following liver transplantation (LTX). Plasma concentrations of Delta-like Canonical notch ligand 1 (DLL1), given as normalized values to the initial plasma levels at day 0 following LTX for patients with bacterial infections (black dots) and without bacterial infection (white dots). Values are presented in percent.

Patients with a bacterial infection within the first 28 days following LTX showed increased plasma levels of DLL1 at all timepoints over the whole observation period compared to patients without microbiological findings, with significant differences on days 0, 1, 2, 7, 14, and 21. Within both groups, DLL1 values were increasing within the first 24 h following LTX. The values in non-infected patients stayed on a stable level until a decrease at day 7 after LTX, whereas the concentrations in infected patients peaked 48 h after surgery and then decreased slowly (Figure 2A, Supplementary Table S2). PCT levels climbed in all patients postoperatively, peaked 24 h later, and decreased afterwards with wide ranges. CRP levels were undulating in all patients on elevated levels. Interleukin (IL)-6 was elevated in all patients directly after LTX and decreased afterwards. PCT levels in bacterial infected patients showed significantly elevated plasma levels on days 7 and 14 following LTX compared to non-infected patients (Figure 2B, Supplementary Table S2). However, nearly all patients exhibited values well above the commonly applied threshold of 2ng/L within the first week following LTX. While CRP did not show a difference but did show a consistent elevation (Figure 2C), IL-6 differed between the groups on day 2 and 14 (Figure 2D). Subsequently performed receiver operating characteristic (ROC) analyses for DLL1 and the three other biomarkers showed congruent values with areas under the curves (AUCs) between 0.608 (0.473–0.742) on d1 and 0.656 (0.529–0.783) on day 14 for DLL1, whereas the three other markers showed lower values most of the time (Supplementary Table S3). Percentage changes of DLL1 appointed to the measured levels on the transplantation day (d0) showed values on the starting levels within the infection group with an increase at the first two days after LTX, whereas patients without an infection showed less of an increase at the first two days after LTX and a halving after 14 days and later on (Figure 3).

**Figure 4:**
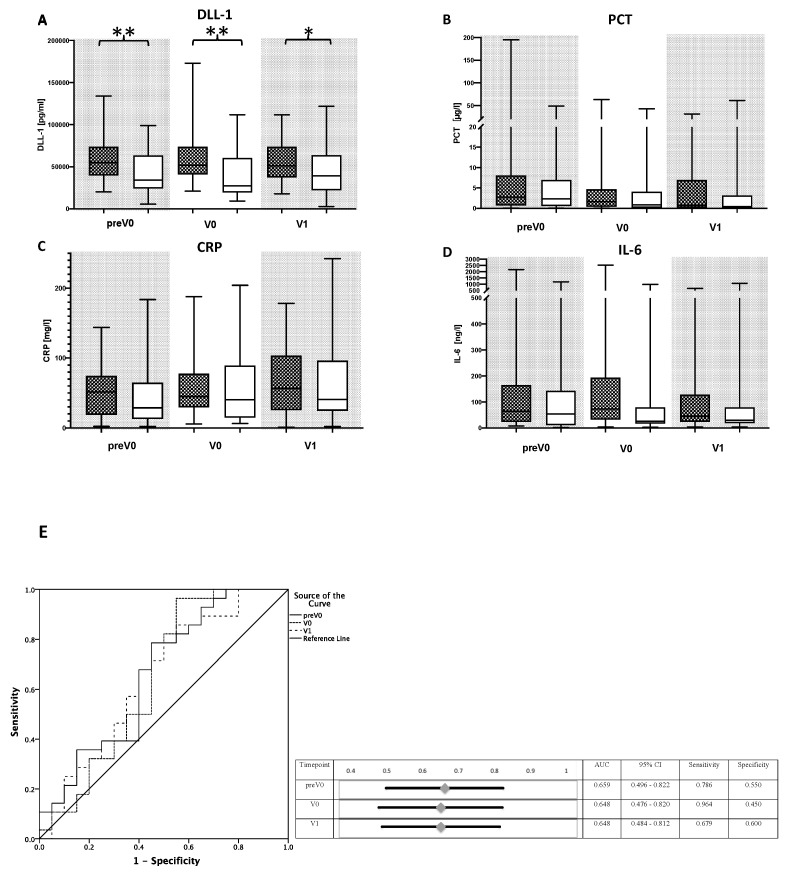
Delta-like Canonical notch ligand 1 (DLL1) for the recognition of bacterial infections matched to the timepoint of the first bacterial findings. Plasma concentrations of (**A**) Delta-like Canonical notch ligand 1 (DLL1), (**B**) procalcitonin (PCT), (**C**) C-reactive protein (CRP), and (**D**) interleukin (IL-)6 were measured in patients following LTX with a bacterial infection (black-checkered box) or without (white box). In patients with a bacterial infection, timepoints were matched to the first bacterial findings, whereas the control group without a bacterial infection was matched to patient´s individual age and sex related to the same timepoints. The following new virtual timepoints were created: first measurement before the first bacterial infection (preV0), the plasma level at time of the bacterial infection (V0), and next following measured plasma level (V1). Data presentation: box plots with median, 25th percentile and 75th percentile in the box, as well as with the 10th and 90th percentiles at the end of the whiskers. Symbols of significance: *p* < 0.05: *, *p* < 0.01 **. (**E**) ROC analysis for DLL1 in bacterial infected vs. uninfected patients regarding the three virtual timepoints preV0, V0, and V1, which are described above. Abbreviations: AUC, area under the curve; CI, confidence interval.

In a subsequent analysis, we adjusted the measurement to the timepoint of first bacterial finding, creating new virtual timepoints (V). Patients with a bacterial infection showed significantly elevated plasma levels of DLL1 at the timepoint before the microbial proof of infection and on the two timepoints after diagnosis (Figure 4A). Subsequently performed ROC analyses revealed an AUC between 0.648 (0.476–0.820) and 0.659 (0.496–0.822) (Figure 4E). In contrast, CRP, PCT, and IL-6 showed no relevant differences in these matching analyses (Figure 4B–D). In a logistic regression model, the combination of DLL1 with PCT or CRP and all three markers together were tested and showed AUCs between 0.731 (0.564–898) at V0 for the combination of DLL1 and PCT and 0.92 (0.974–1.000) for the combination of DLL1, CRP, and PCT at V0 (data not shown).

### 3.3. DLL1 for the Stratification of Patients with High Postoperative Risk

**Figure 5:**
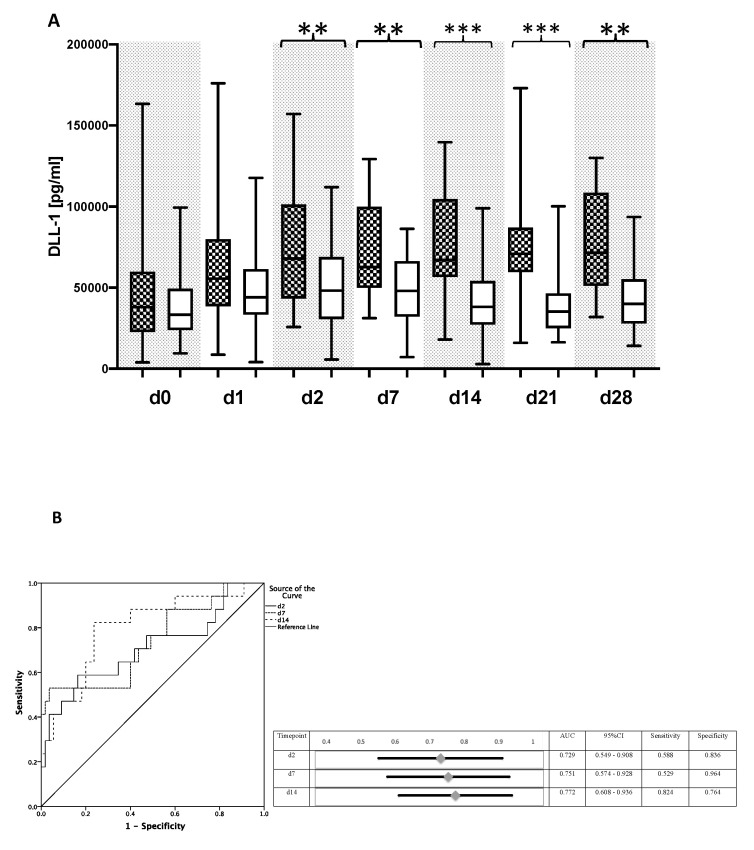
Delta-like Canonical notch ligand 1 (DLL1) for the prediction of a complicated course. (**A**) Plasma concentrations of Delta-like Canonical notch ligand 1 (DLL1) were measured in patients who suffered from two or more complications (rejection, liver failure, acute renal failure, acute lung injury) following LTX or died within 90 days after LTX (black checkered box) and in patients with less or no complications (white box). Plasma samples were collected directly after LTX and within the following 28 days afterwards on the indicated day. Data presentation: box plots with median, 25th percentile and 75th percentile in the box, as well as with the 10th and 90th percentiles at the end of the whiskers. Symbols of significance: *p* < 0.01: **, *p* < 0.001 ***. (**B**) Receiver operating characteristic (ROC) analysis in all patients regarding the value of DLL1 for the prediction of a complicated course on day 2, day 7, and day 14 after LTX. Patients with two or more complications up to day 28 or death within 90 days represented the target group, whereas the remaining patients were defined as controls. Abbreviations: AUC, area under the curve; CI, confidence interval.

Patients with two or more complications showed elevated plasma levels of DLL1 over the whole observation period, with significant differences starting from day 2 (Figure 5A). In contrast, PCT and IL-6 showed no significant differences between the two subgroups, whereas CRP values were significantly increased starting on day 14 in patients with a complicated course. Of interest, median concentrations of DLL1 in the subgroup with a complicated course were higher than those of the subgroup of patients with bacterial infections. As assessed by ROC analyses, DLL1 was able to risk-stratify patients with a complicated course, especially on day 2 with an AUC of 0.871 (CI: 0.773–0.970) (Figure 5B). Even the rate of rejection was significantly higher in the subgroup of patients with a complicated course; significant differences in DLL1 values between patients with or without rejection were not observed.

## 4. Discussion

Within this secondary analysis of a prospective clinical investigation in liver transplanted patients, DLL1 was shown to be a useful option for the detection of bacterial infections in patients following LTX, especially in combination with CRP and PCT. Moreover, it may be used to risk stratify complicated courses within patients after LTX.

Patients following LTX are hallmarked by a high risk for bacterial infections caused by the use of immunosuppressive drugs [18] or, especially in the early period after LTX, by surgical procedures [19]. The appearance of bacterial infections is associated with an increased morbidity and mortality [20]. Therefore, a fast and reliable diagnosis is necessary. However, standard culture-based procedures are associated with relevant weaknesses, such as time duration for results as well as false negative results [21,22]. Molecular approaches, like polymerase chain reaction (PCR)- or next generation-sequencing (NGS)-based methods might be helpful to solve this problem. Unfortunately, available commercial PCR-based kits are described as useful but detect only a limited number of pathogens [23], whereas an NGS-based approach is until now only available in study settings [12].

Using plasmatic biomarkers may help to reduce the diagnostic gap of culture-based methods. Unfortunately, the actually used biomarkers like CRP, PCT, or IL-6 are fare away from being perfect markers [8,24].

In contrast to PCT, CRP, or IL-6, DLL1 in our collective was elevated over a longer time period, which is comparable to the findings in septic patients [11]. Nevertheless, DLL1 plasma values were much higher as compared to those of septic patients [11]. Moreover, by investigating DLL1 levels at the first timepoint of bacterial findings within our collective, we were able to demonstrate within a logistic regression model that DLL1 may give additional value to the routine infectious parameters PCT and CRP. This is comparable to the findings within septic patients [11], in which bacterial findings are mostly detected at sepsis onset and therefore equal to our created virtual timepoint. Since immunosuppressive therapy to prevent rejection largely impacts the adaptive immune cells, especially T-cells, the innate immune system of patients after liver transplantation is not affected, as monocytes have been shown to be an important source of DLL-1 after bacterial stimulation [10,25]. Within daily routine care, this finding may help clinicians to better interpret inconclusive routine infectious parameter findings.

Even the role of DLL1 as Notch signaling molecule is known [26,27], the exactly cellular source is unknown. Endothelial cells as well as monocytes are claimed to secret relevant amounts of DLL1 [28]. Patients following LTX need immunosuppressive drugs in order to avoid an acute rejection [29,30]. Within our collective corticosteroids, mycophenolate mofetil and the calcineurin inhibitors (CNI) cyclosporine or tacrolimus were used for immunosuppression. These drugs are influencing the immune system by blocking T-cell signaling in case of CNI or by inhibiting the chemo-attraction of monocytes in case of mycophenolate [31,32]. Importantly, CNI (especially cyclosporine) activate signal transducers and activators of transcription (STAT) 3 via phosphorylation, which subsequently induce an elevated expression of DLL1 [10,33]. These facts might explain the here presented increase in DLL1 levels following LTX, which is conclusive to findings in patients following heart transplantation [28].

In patients following heart transplantation, the highest levels of DLL1 were described in patients with an acute rejection (median: 26,600 pg/mL; IQR: 23,100–31,000 pg/mL) [28]. Regarding rejections within the presented work, there were no relevant differences between patients with an acute liver graft rejection and without. In contrast, there were higher levels for patients with bacterial infections (e.g., T0: median 38,753 IQR: 30,499–59,827 pg/mL) or complicated courses (e.g., T0: median 47,108 IQR: 37,877–54,932 pg/mL), which has not been described within literature until now.

In infections and rejections following heart transplantation, DLL1 was also described as marker within patients suffering from chronic heart failure or dilatative cardiomyopathy [34,35] as well as non-small cell lung cancer (NSCLC) and chronic obstructive pulmonary disease (COPD) [36]. The described plasma levels of DLL1 in these three studies were much lower as compared to DLL1 levels in our collective. None of our patients suffered from an acute heart failure, a dilatative cardiomyopathy or a NSCLC. Regarding patients with COPD within our collective, we could not observe significant differences between the subgroups. Impaired renal function causes elevated levels of DLL1, as it is cleared by the kidney and therefore accumulates when kidney function is reduced [11]. Within the presented work, no significant differences were shown concerning renal parameters. Neither pre-existing renal impairment, nor acute renal failure differed between patients with a bacterial infection or without. Nevertheless, patients with posttransplant need for dialysis showed significant higher DLL-1 levels. Which might have influenced our results, even less of the half patients with bacterial infection had the need of posttransplant dialysis and the results of the multivariate analysis negotiated a significant influence. Moreover, it is known that renal failure after LTX is a risk factor for a bacteraemia [37] and vice versa that infections after LTX may cause acute renal failure up to the need of a renal replacement therapy [38]. Therefore, our results do not exclude each other, but are more over conclusive.

Despite the solely examination of several complications like graft rejection or acute liver failure did not show significant differences in bacterial infected patients, it might nevertheless influence patient’s outcome. Therefore, it is conclusive, that our patients with several complications had elevated disease severity scores, showed significant elevated CRP-levels starting from day 14 after LTX and died earlier. In patients suffering from two or more complications within the here presented work, DLL1 levels were highly elevated compared to the described plasma levels within heart patients or septic patients [11,34,35]. Moreover, DLL1 levels were also higher compared to those in bacterial infected patients following LTX. The explanation for this fact is certainly multifactorial. First, all complicating factors (acute renal failure, acute liver failure, bacterial infection, acute rejection) can induce elevated levels of DLL1 as described previously [11,28,34,39]. This is especially in line with the finding, that patients with posttransplant need for dialysis had significant elevated DLL-1 levels Second, the combination may trigger an accumulation of DLL1 and might explain exorbitant high levels. Nevertheless, it remains uncertain, which factor is causative and which is only an attending fact.

The secondary analysis presented here is embossed by several limitations. The underlying primary study was performed in terms of a pilot study focusing on fungal infections within a highly selected cohort of patients as well as a limited number of enclosed participants, with enlarged observation periods and a decreasing number of samples at later timepoints, combined with missing pretransplant plasma control samples. Nevertheless, all plasma samples as well as routinely performed microbiological analyses including bacterial, fungal and viral results were available for these secondary analyses. Therefore, the presented results seem to be coherent but need to be reevaluated within a larger prospective clinical study.

## 5. Conclusions

Since bacterial infections in patients after LTX are linked with an increased morbidity, a fast and reliable diagnostic approach is absolutely necessary. Therefore, plasmatic measurements of DLL1 may help the clinician to identify patients with an increased risk for bacterial infections, especially within the first 7 days following LTX. Moreover, it may be used to support the diagnosis of a bacterial infection in case of inconclusive results of routine infection parameters such as CRP and/or PCT, especially in combination with both of them. Besides diagnosis of bacterial infection, elevated levels of DLL1 following LTX may be an indicator for a complicated course within 28 days or death within 90 after LTX.

## Figures and Tables

**Table 1:** Baseline Demographic Data of All Patients and Bacterial Infection Subgroups.

Parameter	Unit	All Patients (*n* = 93)	With Bacterial Infection (*n* = 47)	Without Bacterial Infection (*n* = 46)	*p*-Value for Patients with Bacterial Infection vs. without Bacterial Infection
Male gender		58 (62.3%)	27 (57.5%)	31 (67.4%)	0.219
Age	(years)	52 (42–58)	52 (45–58)	52.5 (40–59)	0.721
BMI	(kg/m^2^)	25.53 (22.99–29.86)	26.45 (23.88–30.03)	24.94 (22.69–29.07)	0.440
MELD score		18.0 (11.0–28.0)	20.0 (13.0–30.0)	14.5 (10.0–22.8)	0.053
Causes of liver cirrhosis					
Alcohol		27 (29.0%)	15 (31.91%)	12 (26.1%)	0.348
Hepatitis B		6 (6.5%)	2 (4.3%)	4 (8.7%)	0.328
Hepatitis C		10 (10.8%)	5 (10.6%)	5 (10.9%)	0.616
HCC		25 (26.9%)	10 (21.3%)	15 (32.6%)	0.159
PSC		16 (17.2%)	9 (19.1%)	7 (15.2%)	0.411
PBC		5 (5.4%)	4 (8.5%)	1 (2.2%)	0.187
NASH		7 (7.5%)	4 (8.5%)	3 (6.5%)	0.512
Others		20 (21.5%)	8 (17.0%)	12 (26.1%)	0.209
Pre-existing MDR colonization		25 (26.9%)	18 (38.3%)	7 (15.2K%)	0.013 *
Need for catecholamines before LTX		3 (3.2%)	2 (4.3%)	1 (2.2%)	0.492
NYHA 0-I		90 (96.8%)	45 (95.7%)	45 (97.8%)	0.508
Diabetes mellitus		18 (19.4%)	12 (25.5%)	6 (13.0%)	0.103
Arterial hypertension		28 (30.1%)	13 (27.6%)	15 (32.6%)	0.384
Coronary heart disease		10 (11.4%)	7 (14.9%)	3 (6.5%)	0.388
Chronic obstructive lung disease		7 (7.5%)	3 (6.4%)	4 (8.7%)	0.450
Smoker		21 (22.5%)	13 (27.7%)	8 (17.4%)	0.189
Pre-existing renal insufficiency		20 (21.5%)	13 (27.7%)	7 (15.2%)	0.400
Pre-existing ARF		10 (10.8%)	8 (17.0%)	2 (4.3%)	0.119
Pre-existing thrombosis		16 (18.2%)	10 (21.3%)	6 (13.0%)	0.269
Neurological disorder		40 (45.5%)	21 (44.7%)	19 (41.3%)	0.228
High-urgency listing		32 (34.4%)	16 (34.0%)	16 (34.8%)	0.557
Re-LTX		16 (17.2%)	10 (21.3%)	6 (13.0%)	0.219
Immunosuppressive medication					
Corticosteroids		93 (100%)	11 (100%)	8 (100%)	
Mycophenolat		92 (98.6%)	47 (100%)	45 (97.8%)	0.495
mofetil					
Ciclosporin		39 (41.9%)	20 (42.6%)	19 (41.3%)	0.535
Tacrolimus		54 (58.1%)	27 (57.4%)	27 (58.7%)	0.535
APACHE II score ^+^		27 (17–32)	26 (18–34)	28 (15–31)	0.252
SOFA score ^+^		13 (7–15)	13 (9–7)	12 (5–15)	0.160
SAPS ^+^		52 (30–69)	56 (3–71)	49 (26–67)	0.254
Duration of mechanical ventilation	(days)	1 (1–4)	2 (1–5)	1 (0–3)	0.035 *
Tracheostomy		11 (11.8%)	8 (17.0%)	3 (6.5%)	0.106
Hospital stay	(days)	1 (1–7)	1 (1–13)	1 (1–1)	0.065
before LTX					
ICU stay	(days)	13 (8–24)	15 (10–36)	11 (7–7)	0.005 **
Hospital stay	(days)	34 (25–52)	43 (29–62)	31 (21–46)	0.012 *
90-day survival		73 (78.5%)	36 (76.6%)	37 (80.4%)	0.422
28-day survival		86 (92.8%)	45 (95.7%)	41 (89.1%)	0.209
TLF after LTX		14 (16.1%)	6 (12.8%)	8 (17.4%)	0.370
ARF after LTX		31 (33.3%)	16 (34.0%)	15 (32.6%)	0.529
ALI after LTX		6 (6.5%)	4 (8.5%)	2 (4.3%)	0.337
Dialysis					
Directly after LTX		6 (6.5%)	5 (10.6%)	1 (2.2%)	0.107
In time course		27 (20%)	20 (42.6%)	7 (15.2%)	0.003 **
Duration of surgery	(min)	347 (289–405)	360 (313–418)	330 (281–401)	0.108
Intraoperative blood loss	(L)	3.0 (1.5–4.4)	3.0 (1.5–5.0)	3.0 (2.3–7.5)	0.292
Rejection		20 (21.5%)	12 (25.6%)	8 (17.4%)	0.241
Perforation of the intestine or stomach		4 (4.3%)	3 (6.4%)	1 (2.2%)	0.317
Stenosis of the bile duct		9 (9.7%)	5 (12.8%)	4 (6.5%)	0.254
Leakage of the bile duct		10 (10.8%)	5 (10.6%)	5 (10.9%)	0.500
Need for surgical intervention		44 (47.3%)	23 (48.9%)	21 (45.7%)	0.456
Vascular complications		13 (14.0%)	5 (10.6%)	8 (17.4%)	0.262
Need for endoscopic diagnostics		13 (14.0%)	10 (21.3%)	3 (6.5%)	0.038 *

Data are presented either as numbers (with the corresponding percentage values) or as median values (with accompanying quartiles Q1–Q3). **Legends:** BMI = body mass index, MELD = model for end-stage liver disease, HCC = hepatocellular carcinoma, PSC = primary sclerosing cholangitis, PBC = primary biliary cirrhosis, NASH = non-alcoholic steatohepatitis, MDR = multidrug-resistant bacteria, NYHA = New York Heart Association Score, ARF = acute renal failure, LTX = liver transplantation, TLF = transplant liver failure; ALI = acute lung insufficiency, APACHE = Acute Physiology And Chronic Health Evaluation, SOFA = Sequential Organ Failure Assessment, SAPS = Simplified Acute Physiology Score, ICU = intensive care unit. ^+^ Calculated at the first day after transplantation. Concerning symbolism and higher orders of significance: *p* < 0.05 *, *p* < 0.01 **.

**Table 2:** Baseline Demographic Data of Complication Subgroups.

Parameter	Unit	With >2 Complications or Dead within 90 Days (*n* = 27)	With <2 Complications (*n* = 66)	*p*-Value for Patients with >2 Complications or Dead within 90 Days vs. <2 Complications
Male gender		18 (66.7%)	40 (60.6%)	0.381
Age	(years)	52 (39–58)	52 (45–58)	0.909
BMI	(kg/m^2^)	25.53 (23.08–30.72)	25.86 (22.99–29.49)	0.024 *
MELD score		20 (14–35)	17 (10–23)	0.406
Causes of liver cirrhosis				
Alcohol		7 (25.61%)	20 (30.3%)	0.438
Hepatitis B		0 (0.0%)	6 (9.1%)	0.119
Hepatitis C		2 (7.4%)	8 (12.1%)	0.399
HCC		3 (11%)	22 (33.3%)	0.022 *
PSC		6 (22.2%)	10 (15.2%)	0.296
PBC		1 (3.7%)	4 (6.1%)	0.546
NASH		4 (14.8%)	3 (4.5%)	0.105
Others		7 (25.9%)	13 (19.7%)	0.343
Pre-existing MDR colonization		9 (33.3%)	16 (24.2%)	0.272
Need for catecholamines before LTX		0 (0.0%)	3 (4.5%)	0.353
NYHA 0-I		27 (100%)	63 (95.5%)	0.353
Diabetes mellitus		5 (18.5%)	13 (19.7%)	0.573
Arterial hypertension		10 (37.0%)	18 (27.3%)	0.254
Coronary heart disease		3 (11.1%)	7 (10.6%)	0.111
Chronic obstructive lung disease		1 (3.7%)	6 (9.1%)	0.344
Smoker		6 (22.2%)	15 (22.7%)	0.551
Pre-existing renal insufficiency		3 (11.1%)	17 (15.8%)	0.111
Pre-existing ARF		2 (7.4%)	8 (12.1%)	0.384
Pre-existing thrombosis		2 (7.4%)	14 (21.2%)	0.151
Neurological disorder		10 (37.0%)	30 (45.5%)	0.311
High-urgency listing		9 (33.3%)	23 (34.8%)	0.544
Re-LTX		4 (14.8%)	12 (18.2%)	0.477
Immunosuppressive medication				
Corticosteroids		27 (100%)	66 (100%)	
Mycophenolat		27 (100%)	65 (90.1%)	0.710
mofetil				
Ciclosporin		12 (44.4%)	27 (40.9%)	0.465
Tacrolimus		15 (55.5%)	39 (59.1%)	0.465
APACHE II score ^+^		29 (21–34)	25 (16–31)	0.593
SOFA score ^+^		13 (11–18)	12 (6 -15)	0.436
SAPS ^+^		61 (43–77)	43 (29–67)	0.359
Duration of mechanical ventilation	(days)	5 (2–14)	1 (1–2)	0.742
Tracheostomy		6 (18.5%)	5 (7.6%)	0.056
Hospital stay	(days)	1 (1–9)	1 (1–6)	0.503
before LTX				
ICU stay	(days)	27 (9–47)	13 (8–17)	0.986
Hospital stay	(days)	44 (26–72)	32 (25–50)	0.452
90-day survival		36 (76.6%)	37 (80.4%)	0.313
28-day survival		25 (92.6%)	61 (92.4%)	0.328
TLF after LTX		3 (11.1%)	11 (16.7%)	0.477
ARF after LTX		9 (33.3%)	22 (33.3%)	0.600
ALI after LTX		3 (11.1%)	3 (4.5%)	0.150
Dialysis				
Directly after LTX		0 (0.0%)	6 (9.1%)	0.119
In time course		8 (29.7%)	19 (28.8%)	0.562
Duration of surgery	(min)	375 (293–420)	346 (293–401)	0.479
Intraoperative blood loss	(L)	2.0 (1.0–3.8)	3.0 (1.5–4.6)	0.026 *
Rejection		11 (40.7%)	9 (13.6%)	0.006 **
Perforation of the intestine or stomach		0 (0.0%)	4 (6.1%)	0.247
Stenosis of the bile duct		3 (11.1%)	6 (9.1%)	0.516
Leakage of the bile duct		2 (7.4%)	8 (12.1%)	0.475
Need for surgical intervention		14 (51.9%)	30 (45.5%)	0.370
Vascular complications		2 (7.4%)	11 (16.7%)	0.204
Need for endoscopic diagnostics		6 (22.2%)	7 (10.6%)	0.129

Data are presented either as number (with the corresponding percentage value) or as median (with accompanying quartiles (Q1–Q3). **Legends:** BMI = body mass index, MELD = model of end-stage liver disease, HCC = hepatocellular carcinoma, PSC = primary sclerosing cholangitis, PBC = primary biliary cirrhosis, NASH = non-alcoholic steatohepatitis, MDR = multidrug-resistant bacteria, NYHA = New York Heart Association Score, ARF = acute renal failure, LTX = liver transplantation, TLF = transplant liver failure, ALI = acute lung insufficiency, APACHE = Acute Physiology And Chronic Health Evaluation, SOFA = Sequential Organ Failure Assessment, SAPS = Simplified Acute Physiology Score, ICU = intensive care unit. ^+^ Calculated at the first day after transplantation. Concerning symbolism and higher orders of significance: *p* < 0.05 *, *p* < 0.01 **.

**Table 3:** Risk Factors for the Occurrence of Bacterial Infections Following LTX.

Variable	Univariate Analysis with 95% CI	*p*-Value	Multivariate Analysis with 95% CI	*p*-Value
DLL-1 d2	3.03 (1.26–7.28)	0.010 **	1.76 (0.57–5.42)	0.323
Pretransplant diabetes mellitus	2.74 (0.87–8.55)	0.103	3.20 (0.88–11.57)	0.075
Dialysis following LTX	4.02 (1.49–10.48)	0.003 **	2.40 (0.62–9.19)	0.200
MELD	1.03 (0.99–1.08)	0.053	1.02 (0.97–1.07)	0.306
Duration of surgery	1.03 (0.99–1.08)	0.108	1.00 (0.99–1.00)	0.573
Pretransplant MDR	3.36 (1.24–9.14)	0.013 *	2.26 (0.76–6.74)	0.141

**Legends:** DLL-1 = Delta-like canonical Notch ligand 1, MDR = multidrug-resistant bacteria, LTX = liver transplantation. Data given as odds ratio with 95% confidence interval. Symbols of significance: *p* < 0.05 *, *p* < 0.01 **.

**Table 4:** Risk Factors for the Occurrence of >2 Complications within 28 Days after LTX or Dead within 90 Days.

Variable	Univariate Analysis with 95% CI	*p*-Value	Multivariate Analysis with 95% CI	*p*-Value
DLL d2	4.40 (1.55–12.41)	0.05 *	2.11 (0.36–12.38)	0.407
Dialysis following LTX	13.57 (4.60–40.03)	0.001 ***	6.87 (1.28–34.20)	0.018 *
HCC	0.25 (0.06–0.92)	0.022 *	0.00 (0.00–0.00)	0.998
Rejection	4.76 (1.67–13.47)	0.006 **	0.00 (0.00–0.00)	0.998
BMI	4.72 (1.65–13.47)	0.024 *	0.90 (0.78–1.04)	0.158
Intraoperative blood loss	1.04 (0.94–1.16)	0.026 *	0.99 (0.86–1.12)	0.880

**Legends:** DLL-1 =Delta-like canonical Notch ligand 1, LTX = liver transplantation, BMI = body mass index. Data given as odds ratio with 95% confidence interval. Symbols of significance: *p* < 0.05 *, *p* < 0.01 **, *p* < 0.001 ***.

## Supplementary Materials

The following are available online at https://www.mdpi.com/2075-4418/10/11/894/s1, Table S1: Influence of significant factors within the univariate analysis on DLL-1 plasma levels, Table S2: Plasma values of the infection parameters, Table S3: Area under the curve (AUC) values of different infection parameters regarding the prediction of a bacterial infection within 28days following LTX, Statement S1: STROBE Statement—checklist of items that should be included in reports of observational studies
